# Claudin Proteins: Their Potential Role in Obesity and Adipose Tissue Signaling, Physiology and Disease

**DOI:** 10.3390/nu17162611

**Published:** 2025-08-12

**Authors:** Pablo Fernández-García, Francesc Villarroya, David Sánchez-Infantes, Patricia Corrales

**Affiliations:** 1 Area of Biochemistry and Molecular Biology and Genetics, Department of Basic Health Sciences, Campus Alcorcón, Rey Juan Carlos University (URJC), 28922 Alcorcón, Spain; pablo.fernandezga@urjc.es; 2 Centro de Investigación Biomédica en Red de Fisiopatología de la Obesidad y Nutrición (CIBEROBN), Instituto de Salud Carlos III, 28029 Madrid, Spain; fvillarroya@ub.edu; 3 Department of Biochemistry and Molecular Biomedicine, Institute of Biomedicine, University of Barcelona, 08028 Barcelona, Spain

**Keywords:** claudins, adipose tissue, cell signaling, obesity

## Abstract

Obesity is one of the most challenging metabolic disorders affecting more than 800 million people around the world, according to the World Health Organization (WHO). In recent years, our knowledge and understanding of this multifactorial disease have been exponentially increasing, and many studies have been focusing on one of the main organs affected by obesity: adipose tissue (AT). It is known that AT undergoes remodeling due to the abnormal fat accumulation that accompanies obesity, characterized by increased immune cell infiltration, extracellular matrix (ECM) overproduction, and decreased adipogenesis, among others. Few studies have focused on adipocyte intercommunication, even though it is essential for AT homeostasis and function. In this context, GAP junction, adherens junction, and tight junction proteins can be found in these depots. In some cases, their function is well established, but in most cases it remains unknown. Claudins are the main proteins that form tight junctions (TJs), and, in recent years, studies have revealed a more extensive role of claudin proteins in intracellular signaling and control of a wide set of biological processes. This review aims to gather the main scientific evidence on the role of claudins in cell signaling, as well as what is known about these proteins in the field of obesity and adipose tissue physiology.

## 1. Introduction

Obesity is the result of abnormal and/or excessive fat accumulation in the body and represents a health risk. According to the World Health Organization (WHO), it is the most prevalent chronic metabolic disorder. In 2022, the prevalence of obesity was higher than that of being underweight in 89% of countries for women and 73% for men [1]. Moreover, in that same year, obesity in school-aged children and adolescents was more prevalent than thinness in 67% of countries for girls and 63% for boys [1]. Obesity is a major risk factor for several chronic diseases, including cardiovascular diseases, musculoskeletal disorders, type 2 diabetes (T2D), fatty liver, and even some types of cancers, thus reducing life expectancy [2]. In genetically susceptible individuals, obesity results in a positive energy imbalance facilitated by overnutrition and a sedentary lifestyle [3]. The pathological obese state can accelerate adipose tissue (AT) remodeling to respond to alterations in excessive fat in the whole body. The AT remodeling process accompanying obesity is characterized by decreased adipogenesis, extracellular matrix (ECM) overproduction, angiogenic remodeling, and increased immune cell infiltration into AT, all ultimately resulting in unhealthy systemic consequences [4].

**Figure 1:**
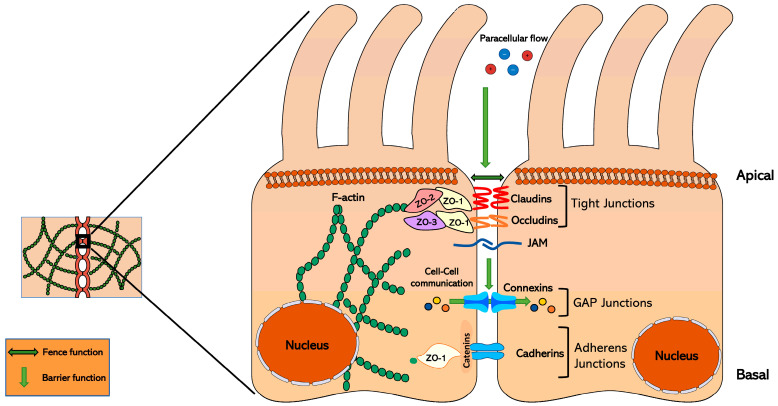
Schematic representation of the tight junction structure in epithelial cells. Claudins control the paracellular flow of water, ions, and other small molecules between cells. They are in contact with zonula occludens (ZO) proteins, forming a scaffolding structure that allows them to interact with different cell signaling pathways. On the other hand, GAP junctions allow communication between adjacent cells, together with adherens junctions, which keep the cells together.

Nowadays, there is evidence that inter-adipocyte communication through cell-to-cell contacts, adipokines, and/or extracellular vesicles is important for AT biology and remodeling [5]. Over the last decades, junctions between cells and their molecular components have been increasingly identified as essential for cell adhesion and permeability in tissues and organs. Among the contacts described between adipocytes, GAP junctions are the most important as they allow intercellular communication [6,7]. In this context, GAP junctions have been colocalized in other tissues together with other junction proteins, such as claudins (present in the tight junction [TJ] between cells) [8]. However, the role of TJs has not yet been analyzed in depth in AT or in an obesity context, thus highlighting that this field of study could be a key focus for advancing knowledge of this multifactorial illness. There are three main intercellular junctions (Figure 1): TJs, adherens junctions (AJs), and GAP junctions [9]. These complexes differ in the type of proteins that make them up, with the most abundant being junctional adhesion molecules (JAMs), claudins and occludins (both TJs), cadherins (AJs), and connexins (GAPs) [10,11]. TJs can be defined as cell–cell contacts that partially seal the space between cells, creating selective permeability barriers and/or channels [12] (Figure 1). They are responsible for paracellular permeability (gate function) and contribute to the maintenance of cell membrane polarity, restricting the diffusion of lipids outside the plasma membrane and the intermixing of its apical and basolateral domains (fence function) (Figure 1). The study of non-canonical functions has shown that TJs can bind to similar neighboring junctions to provide key input between proximal cells. In this context, TJs can provide key input from adjacent cells, which translates into signaling cascades through the formation of a signalosome structure with cytoplasmic scaffolding proteins (such as zonula occludens [ZO]-1, -2, and -3) and the cytoskeleton (Figure 1), having a key role in determining different cell functions [13]. The TJ gate function is allowed by the formation of small claudin channels that can be anion/cation and/or water selective, depending on the charge or the size of the molecules [10]. Recent studies have been revealing a more extensive role for junction components, such as claudins, in the control of a wide set of biological processes, including intracellular signaling [14,15,16]. There is increasing awareness that claudins may be key actors in complex processes of metabolic regulation, whose disturbances are associated with obesity and related pathologies.

The study of claudins and their association with cell signaling has been expanding, and nowadays they are considered downstream and upstream signaling members of several cellular pathways [17,18,19]. This fact reveals that some claudin proteins have non-canonical functions, primarily unrelated to their role as TJ components, with a significant role in tissue homeostasis, such as the regulation of metabolic reprogramming and the alteration of cell plasticity [20]. Despite scattered reports on the non-canonical functions of claudins and their role in cell signaling, there are currently very few reviews compiling available information on the potential role of claudins in obesity and other metabolic alterations, either via their role in TJs or through non-canonical actions. In this review, we discuss the potential role of claudin proteins in cell signaling, AT functionality, and obesity.

From this point onwards, the nomenclature used to refer to the different claudins will be CLDNs for proteins and *CLDNs* for genes.

## 2. Tight Junction Proteins: Junctional Adhesion Molecule, Occludin, and Claudin Structures and Functions

TJs are constituted by a combination of several molecules whose interaction and individual functions allow the correct operation of the TJ structure. Among these proteins, JAMs, occludins, and claudins can be found.

### 2.1. Junctional Adhesion Molecules and Occludins

JAMs and occludins are claudin-independent molecules known to be TJ-associated MAL and related proteins for vesicle trafficking and membrane link (MARVEL) domain-containing proteins [10]. MARVEL proteins contain four transmembrane domains (tetraspan proteins), which form the conserved MARVEL domain [21]. JAMs are found in the TJ strands of epithelial and endothelial cells, and they have a key role in the configuration of TJs and epithelial polarity, along with claudins [11,22]. The first JAM protein was identified in 1998, and it is encoded by an immunoglobulin gene superfamily member consisting of two V-type Ig domains [23]. Nowadays, it is known that JAMs are immunoglobulin superfamily (IgSF) proteins specific to cell junctions, present also on the surface of immune cells [24].

On the other hand, occludins were the first transmembrane molecules described as a TJ-specific protein [25]. However, the role of occludins within TJs has been widely discussed over the years. Since its discovery in 1993 [26], further studies showed that this protein is not a relevant player in TJ formation [21] and that general occludin gene knockout in mice does not affect the development of TJs in the intestine, liver, and kidney [27].

### 2.2. Claudins

**Figure 2:**
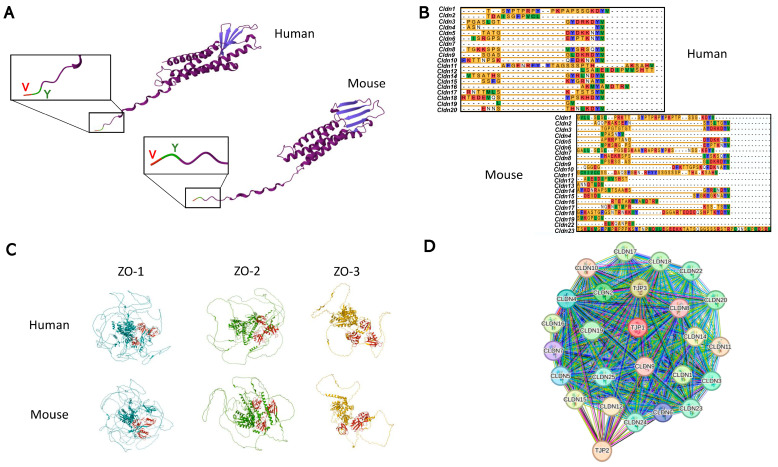
Protein structure of claudins and ZO in humans and mice. (**A**) The protein structure of the CLDN1 protein in humans and mice, indicating the PDZ-binding motif located in the -COOH ending, formed by the Tyr–Valine (YV) amino acids. Obtained using AlphaFold^®^. (**B**) The amino acid sequence of the main claudin proteins in humans and mice, where the conserved PDZ-binding motif (YV) can be observed. Obtained using UniProt^®^ alignment (Clustal Highlight). (**C**) The structure of ZO proteins in humans and mice, highlighting in red the PDZ domains that allow them to interact with claudin proteins. Obtained using AlphaFold^®^. (**D**) String network showing the interconnection between all claudin proteins in humans, with the scaffolding proteins ZO-1, -2, and -3 (*TJP1*, *2*, and *3*). Obtained using STRING^®^.

Claudins are the most important structural component of TJs, so much so that the expression of these proteins is enough to reconstitute TJs in cells that lack these structures [25]. Currently, 27 claudin genes (*CLDN1-27*) have been identified in mammals, except for *CLDN13*, which is only present in rodents [28]. Claudins in humans and mice are made up of 207–305 amino acids, with an average molecular mass of 21–34 kDa [28]. They are therefore a group of tetraspan, small, simple-structured proteins with the additional feature of having a COOH-terminal PDZ-binding motif, which allows them to interact with scaffolding proteins containing a PDZ domain (Figure 2A). This terminal PDZ-binding motif domain (Tyr-Val, YV) is essential for interaction with the scaffold protein ZO [29], allowing claudins to indirectly communicate with the cell’s actin filaments and other signaling proteins (Figure 2A). Moreover, this PDZ domain is highly preserved in almost all claudins both in humans and mice (Figure 2B). PDZ proteins contain different PDZ domains, which are a family of domains with low sequence identity but evolutionarily conserved allosteric residues that allow them to interact with many other proteins besides claudins [30]. ZO proteins (encoded by *TJP1*, *2*, and *3*) have been described to form part of the family of PDZ-domain proteins (Figure 2C) and can recruit other regulatory players (GTPases, kinases, phosphatases, and transcriptional factors) to TJ complexes. As a result, a protein scaffold is formed, which allows the assembly of more complex structures, such as signaling nodes [31].

As mentioned before, the role of TJs is to act as a barrier or channel for different molecules. For this reason, claudins have a key role in determining and regulating paracellular permeability as they control the perm-selectivity of TJs, allowing cation, anion, and/or water permeability via the formation of regulated barriers and channels [14,32,33] (Table 1). The barrier function has been studied by using several knockout (KO) and/or knockdown (KD) animal models. For instance, *Cldn1*-KO mice lose the TJ barrier to water and macromolecules, causing death due to dehydration in the neonatal period [34]. Also, in the skin of mice, *Cldn1*-KO/KD causes lethal dehydration and atopic dermatitis, respectively [14], as a consequence of the loss of the barrier structure. However, it has been recently observed that the lethal effect of CLDN1 deletion may also involve the lack of other TJ proteins, such as CLDN4 and ZO-1 [35], thus leaving the specific role of CLDN1 in this fatal outcome as an open question [14].

The barrier function is also essential for the correct function of specialized structures like the blood–brain barrier (BBB). CLDN3 and CLDN5 are expressed in many epithelia, mainly acting as barrier-forming TJ proteins, with CLDN5 being the most abundant protein in endothelial TJs [28]. In the BBB, *CLDN3*-KO and *CLDN5*-KO generate barrier dysfunction that is important in the development of multiple sclerosis, brain inflammation, seizures, and schizophrenia [14].

On the contrary, CLDN2, 10, 15, 16, and 19 are significant in channel formation (Table 1). The loss of these proteins results in disorders such as renal hypoxia, malnutrition due to intestinal malabsorption, or renal absorption deficiency [14]. In fact, CLDN2 and CLDN15 create paracellular channels that not only allow the flow of cations, but also of water [36,37].

Some claudins act both as a cation barrier and an anion-selective channel according to the cell type, as in the case of CLDN8 [38]. Also, CLDN4, expressed together with CLDN8, generates an anion-selective channel; however, CLDN4 on its own affects Na^+^ permeability [32]. Claudin proteins are also very sensitive to the extracellular levels of other ions, such as Ca^2+^. Studies have shown that Ca^2+^ can act as a regulator of TJ protein (including claudins) assembly and how they respond to extracellular stimuli [39]. Moreover, extracellular Ca^2+^ fluctuations affect the structure and binding activity of claudins through JAM-A [39]. The fact that claudins are so important for regulating cation and anion flow highlights that they play a pivotal role in tissue and cellular osmotic regulation and homeostasis [40,41].

As mentioned above for CLDN4 and CLDN8, some claudins need to be expressed together to perform their function correctly, as these proteins are highly interconnected between themselves and with the *TJP* genes, given the direct interaction of CLDNs with ZO proteins (Figure 2D). Regarding this concept, CLDN16 and CLDN19 also need to interact to be inserted into the TJ complex and be fully functional [42]. Furthermore, the interaction between different claudins can determine not only their function but also their location [28].

In recent years, the study of different claudin isoforms has started to catch the scientific eye. CLDN10 presents different isoforms, mainly CLDN10a and CLDN10b. While CLDN10a expression is specific to the kidney and creates anion-selective channels impermeable to water, CLDN10b is ubiquitously expressed and creates cation-selective channels [43]. Moreover, CLDN10 exhibits more spliced isoforms, which determine their location in the TJ structure or endoplasmic reticulum [44]. In this regard, it has been described that *CLDN5* alleles encode proteins with different open reading frames, and with diverse locations, the long form being positioned in intracellular compartments and the short form in TJs [45]. Altogether, these data suggest that there may be different, as yet unidentified, isoforms of each claudin protein, which provide specific cytoplasmic, organelle, or membrane locations and, therefore, diverse functions.

CLDNs could also form part of the intercellular communication between adjacent cells. The dynamic regulation of claudins is mainly through endocytosis [46]. Specifically, this endocytosis process occurs via the “cell-eat-cell” model, where the whole TJ structure is internalized, taking with it portions of the adjacent cell [46,47]. Considering that CLDN endocytosis occurs by internalizing the whole TJ complex, it could be hypothesized that the signaling hub from the TJs in the adjacent cell could also be in the endocytosed vesicle, giving rise to TJs as intercellular communicators. Based on this claudin endocytosis mechanism, claudins may also be present in intracellular membranes that were once part of the plasma membrane. Mitochondria, for instance, have an inner membrane thought to be a remnant of their bacterial origin, while the outer membrane is hypothesized to be of eukaryotic origin, derived from the vesicle that surrounded the mitochondrial ancestor [48]. In this context, in 2021, CLDN5 was reported to localize at the external mitochondrial membrane in murine cardiomyocytes, playing an important role in mitochondrial fission [49]. The current knowledge regarding TJs gives rise to the study of their components in a more in-depth manner. Specifically, claudins have proven to be the most interesting TJ molecules given their variety of types and functions.

## 3. Claudins and Cell Signaling

In addition to their canonical function in structural cell interactions, the role of TJs and their protein components in cell signaling pathways has also been highly studied in recent years, and they have been established as signaling hubs by experts in this field of study [50]. Claudins play a role in diverse pathologies by interacting with different cellular pathways (Table 2).

### Signaling Hub Locations

Moreover, claudins have been found in different compartments of the cell, not just in TJ complexes but also in the basal or basolateral membrane, cytosol, and even the nucleus [18]. Besides these locations, recent studies have also found exposed claudins in the membrane of the cell, outside the TJ space, with an important interaction with the ECM [17,51,52]. Located within TJs, some of these proteins can interact with multiple signaling pathways, acting as signaling mediators or as final target molecules of specific routes. It has been postulated that claudins participate in cell signaling through signaling hubs-signalosomes, which allow the interaction between these proteins, scaffolding PDZ-domain containing proteins, and the cytoskeleton of the cells [13]. There is a bidirectional signaling flow within TJs: on the one hand, signals from the cell toward TJs guide their assembly and function. On the other hand, signals can be transmitted from TJs to the cell to regulate gene expression [31].

Claudins are also relevant proteins in terms of signaling when located at other sites in the cell. In this sense, basolateral claudins have been proposed as signaling hubs or clusters with the capability of integrating, encoding, and transporting information inside the cell [16]. CLDN1 and CLDN7 can interact and bind to EpCAM (an epithelial cell adhesion molecule), creating a functional signaling hub in the basolateral membrane of intestinal cells [53]. Additionally, to stabilize cell/matrix interactions, the location of some claudins, such as CLDN1, CLDN2, and CLDN7, in the basal membrane of epithelial cells is needed, allowing the regulation of cell/ECM interactions by interacting with integrin molecules via integrin–FAK signaling [16]. Specifically, basal CLDN7 can interact with intestinal integrins to potentially regulate the gene expression of other TJ-associated claudins [16]. CLDN7 has also been proposed as a controller of Wnt/β-catenin signaling in intestinal epithelial stem cells, together with CLDN5, which is also known to upregulate the Wnt pathway in podocytes and brain endothelial cells [54,55].

There is evidence of the presence of claudins inside the nucleus of cells, with CLDN1 being the first human claudin found to be located inside the nucleus of tissues from patients with colon cancer [56]. In this study, the presence of this protein was detected in the nucleus, also finding that healthy human colonic mucosa CLDN1 crossed the basolateral membrane but did not enter the nucleus of the cell [56]. This discovery opens the door to the notion that the localization of the protein determines its function and participation in one or another signaling pathway.

The cytoplasmic domain of claudins contains the binding motif that allows claudins to propagate intracellular signals; however, it is not yet well defined how this signaling can reach the nucleus and regulate the expression of different genes [57]. In this regard, different claudins have been identified in transcription factor signaling cascades [57]. The transcription factors Slug and Snail have been found to act as repressors of *CLDN1* expression in epithelial cells from human breast cancer, affecting tissue permeability and highlighting *CLDN1* as a downstream target gene of the Snail family of transcription factors [58]. Another way of regulating gene expression is through the PI3K/AKT axis. CLDN6 regulates nuclear receptor activity by recruiting Src-family kinases (SFKs), whose activation stimulates downstream PI3K/AKT signaling, leading to RARγ and ERα nuclear receptor activation [59].

## 4. The Potential Role of Claudins in Obesity and Adipose Tissue Physiology and Disease

Obesity is characterized by an increase in AT and its redistribution. AT is a dynamic organ whose function is to store and release energy, sense nutrients, regulate body temperature, and, as an endocrine organ, release hormones, chemokines, and cytokines that modulate food intake, insulin sensitivity, and inflammation [60]. Under healthy conditions, the main function of AT is lipid storage as triglycerides (TGs) in lipid droplets. Nevertheless, the storage capacity of AT can be exceeded beyond an individualized threshold, and lipids can act as toxic lipid species due to their accumulation in other metabolic peripheral tissues, such as the liver, muscle, kidney, and/or pancreas (lipotoxicity), and give rise to metabolic abnormalities [61]. Conversely, when exposed to long-term energy surplus, AT expands by increasing the size of the adipocytes (hypertrophy) and/or increasing the number of new adipocytes (hyperplasia). In lean individuals, this adjustment copes with the demand for lipid storage, and AT presents a phenotype with many small adipocytes reflecting a healthy metabolic profile [62]. In contrast, in patients who live with obesity, AT exhibits a hypertrophied phenotype due to a failure to differentiate new adipocytes and a reduction in adipocyte turnover, i.e., adipocyte birth/death rate correlating with AT dysfunction and insulin resistance (IR) [62]. In this context, this review aims to thoroughly compile the available evidence on the role of claudin proteins in adipose tissue physiology and disease.

### 4.1. Claudins Within the Adipose Tissue

Recent studies on AT have been focusing on several junction proteins, and have revealed that GAP junctions have important roles in adipocyte communication through connexin proteins, such as connexin-43 (Cx43) [6,63]. However, other membrane components have recently emerged as important players in AT physiology: integrins [64]. Active integrins, such as β1-integrin or Kindlin-2, play a key role in the regulation of insulin sensitivity in AT through the establishment of integrin–ECM interactions [64]. Moreover, integrins in endothelial cells have been described to directly interact with various claudin proteins [65,66]. Furthermore, CLDN2 promotes the generation of integrin complexes [67], while CLDN7 interacts with integrin signaling to suppress cell proliferation [68]. Altogether, these findings are opening the door to the hypothesis that claudins may also have a role in the function of integrins within AT.

**Figure 3:**
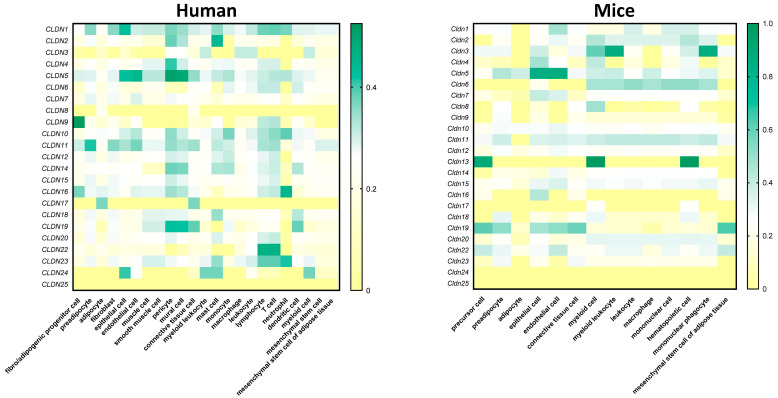
Heatmap of the claudin proteins in human adipose tissue. The gene expression of the different claudins in humans and mice. Data obtained from Cellxgene (https://cellxgene.cziscience.com/; accessed on 6 May 2025). In humans, claudins are present in a greater number of cell types in AT than in mice, thus showing the difference between the animal model and human physiology.

On the other hand, many public databases contain transcriptomic data at the single-cell level, showing that claudins are present in several cell types within AT. At *Cellxgene* (https://cellxgene.cziscience.com/; accessed on 6 May 2025)—a web-based database on public gene expression datasets—claudins can be found expressed at different levels in many cell types within human and mouse AT (Figure 3). Furthermore, Emon et al. and Zhong et al. have published several databases where claudin expression can be found in diverse AT cells (https://singlecell.broadinstitute.org/single_cell/study/SCP1376/a-single-cell-atlas-of-human-and-mouse-white-adipose-tissue; www.adiposetissue.org; accessed on 6 May 2025) [69,70], even though the role of many of them in AT biology has not been studied yet. However, it is necessary to consider the difference in claudin expression patterns between animal models and humans, emphasizing that the results obtained in the former should be meticulously studied in humans (Figure 3). In humans (Figure 3), the claudins most expressed in fatty depots are: *CLDN1* in epithelial and immune cells; *CLDN2* in mast cells; *CLDN5* in endothelial cells, pericytes, and mural cells; *CLDN9* in fibro/adipogenic progenitor cells; *CLDN10* in immune cells; *CLDN11* in preadipocytes; *CDLN16* in neutrophils; *CLDN22* in lymphocytes and T cells; and *CLDN23* neutrophils. On the contrary, in mice, the most expressed claudins in AT are: *Cldn3* in myeloid leukocytes and mononuclear phagocytes; *Cldn5* in epithelial and endothelial cells; and *Cldn13* in precursor, myeloid, and hematopoietic cells (Figure 3). Interestingly, *CLDN5* appears to be highly expressed in endothelial cells both in humans and mice. These findings within AT are consistent with current knowledge about this claudin protein, as it is expressed in endothelial cells in other tissues [71,72,73,74]. Given this evidence, CLDN5 may also have an important role in the already studied interplay between AT and endothelial cells [75,76]. Furthermore, single-nucleus RNA sequencing has recently revealed that endothelial cells in mice are involved in the development of obesity [77], thus highlighting the importance of studying the role of CLDN5 in the context of AT and obesity. It is also worth noting that the expression datasets suggest a more important role of claudins in humans, as they are expressed in a greater number of different cell types (Figure 3). Nevertheless, current research has not yet addressed the current gap between the known presence of claudins within AT and their possible roles in its physiology. Studies based on knockout, knockdown, and even overexpression experiments in vitro and in vivo should be proposed to validate the function of claudin proteins among the diverse AT cell populations.

However, the presence of ACAM (adipocyte adhesion molecule, also known as CLMP) has been reported in AT. This is a cell adhesion molecule implicated in adipocyte maturation and the development of obesity [78]. It has also been determined that ACAM possesses anti-obesity effects by modulating cell adhesion and actin function [79]. Moreover, the presence of this protein in AT is quite interesting, as it has been described to be a component of TJs in epithelial cells, co-localizing with the protein ZO-1 [80]. This issue makes ACAM the first TJ protein ever to be identified in AT. Recently, the TJ proteins CLDN5, occludins, and JAM-1 were reported to be present in the subcutaneous adipose depot of humans [81]. In this study, *CLDN5*, *OCLN*, and *JAM-1* expression were found to be downregulated in subcutaneous fat microvessels incubated with trimethylamine N-oxide (TMAO), a gut-microbiota metabolite that permeates the gut–blood barrier. Specifically, immunofluorescence staining allowed the co-localization of these proteins with CD31 (an endothelial marker) [81]. These data point toward claudins and other TJ proteins as important players in gut barrier dysfunction, a pathology that has been previously related to obesity [82,83].

**Figure 4:**
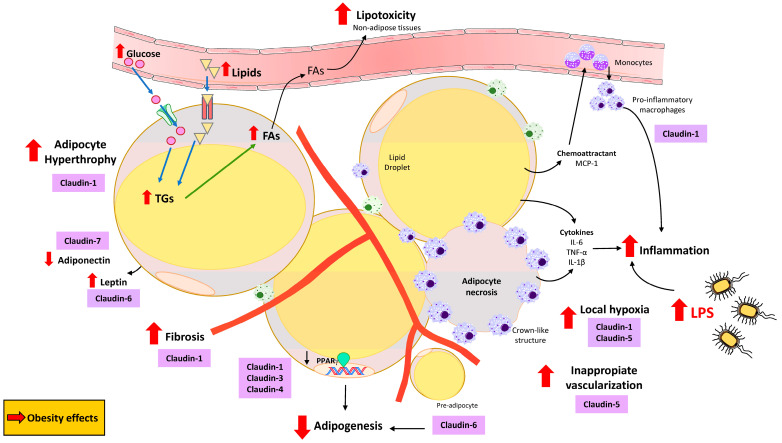
Proposed roles of claudins in adipose tissue physiopathology. Claudins have been suggested to play important roles in obesity, e.g., in the remodeling process (hyperplasia), lipotoxicity, or inflammation. FAs: fatty acids. LPSs: lipopolysaccharides. TGs: Triglycerides.

It has also been proposed that obesity induces changes in tissue permeability via claudin phosphorylation in a process known as “claudin switching” [84,85], accompanied by barrier function deregulation in several organs, even though their findings were not in AT itself. Specifically, restructuring of TJs was observed in the intestinal epithelium of mice fed a high-fat diet (HFD) and in intestinal epithelial cells exposed to leptin in vitro. Claudin switching has been observed in epithelial cells from the gastrointestinal tract, where TJ components are highly dynamic and can undergo remodeling without changes in their structure [86]. This process can be triggered by external stimuli, such as proinflammatory cytokines or growth factors [86]. In HFD-fed mice, HFD-induced obesity has been shown to significantly restructure TJ components in intestinal epithelium [84]. Moreover, the expression of other TJ components does not seem to be affected in mice on an HFD, thus proposing claudin switching as the main factor responsible for TJ remodeling in obesity [84,85]. Moreover, claudin switching has also been proposed as a key process in local and systemic immune homeostasis by possibly compromising the epithelium in the intestine under inflammatory conditions [85]. These findings suggest that HFD-induced obesity can lead to important changes in gut permeability, barrier properties, and ion homeostasis, thus predisposing people living with obesity to many pathological conditions [84]. Furthermore, adipose-produced proinflammatory cytokines may have a potential role not only in the previously proposed intestinal claudin switch but also locally in AT. In obesity, and specifically HFD-induced obesity, the involvement of lipopolysaccharides (LPSs) has been widely studied. In line with this, it has been established that LPSs are involved in the development of obesity and play an important role in lipid delivery and storage in the adipose depots [87]. In fact, LPSs have a direct impact on AT physiology by affecting the size and inflammatory status of adipocyte [87,88] s. In this context, some claudin proteins are also affected by LPSs, such as CLDN1, CLDN3, CLDN4, and CLDN7 [89]. Moreover, betaine treatment has been shown to rescue *CLDN1* downregulation in the damaged intestinal barrier [90]. All these changes in claudins and their effects on AT (Figure 4) highlight that the effect of obesity on claudins could be overcome by pharmacological treatments.

Accordingly, mesenteric AT hyperplasia has been found to exhibit decreased levels of TJ proteins, including CLDN1 [91]. These findings contrast with the effects observed under obesity conditions, where AT displays a hypertrophied phenotype and claudin expression may be excessively increased, potentially contributing to AT dysfunction (Figure 4). Moreover, increased levels of leptin and CLDN6 have been described in AT from rodents fed an HFD [92] (Figure 4).

In the obesogenic condition, *CLDN6* is differentially expressed in fat depots of mice fed an HFD compared with a normal diet, interfering with adipogenesis and fat deposition [92]. In this respect, it is well known that the decreased expression of peroxisome proliferator-activated receptor gamma (PPARγ) inhibits adipogenesis, compensated by AT hypertrophy in obesity, increases ectopic fat accumulation, and increases the risk of IR [93]. Moreover, it has been reported that a reduction in the differentiation capacity of preadipocytes in obesity [62], which may occur as a consequence of PPARγ inhibition in preadipocytes, leads to decreased adipogenesis [94] (Figure 4). There is no information about how PPARγ could regulate the expression of specific TJs in visceral AT (VAT) and subcutaneous AT (SAT); however, *CLDN1* expression was reported to be related to PPARγ in mesenteric fat hyperplasia [91]. Furthermore, it was recently documented that PPARγ plays a crucial role in *CLDN3* and *CLDN4* expression in the intestinal barrier [95], suggesting a possible non-canonical role of claudins in AT adipogenesis in preadipocytes (Figure 4). In fact, one study performed on 3T3-L1 cells reported that when *CLDN6* expression is knocked down, adipogenesis is reduced, indicating that CLDN6 might be another important factor related to adipocyte differentiation [92]. This study is significant as, for the first time, it reported that the expression pattern of a claudin protein is affected in AT cells, thus suggesting that these proteins are present in adipocytes, playing a role in adipogenesis and adipocyte biology. Nevertheless, this study was performed in immortalized cells, and the role of CLDN6 in adipogenesis has not been confirmed yet in in vivo models. It is also important to note that there is no further information regarding how different types of claudins could impact AT adipogenesis; further studies should be carried out.

In the setting of decreased PPARγ expression in obesity, the typical pattern observed includes limiting further AT expansion and dysregulating adipokine production and release patterns, including low levels of adiponectin and high levels of leptin [96]. With this consideration in mind, favorable effects of CLDN7 on adiponectin levels have been identified, along with a positive impact on AT function after an intense lifestyle intervention in patients living with overweight/obesity [97] (Figure 4). These results highlight the importance of the non-canonical function of claudins related to adipokines and AT functionality.

Becoming obese requires AT to be able to expand; for this reason, it is necessary to remodel the ECM and grow vessels to provide oxygenation and optimize nutrient mobilization [98]. In obese AT, collagens can accumulate around the adipocytes to form pericellular fibrosis, or collagen fibers can be organized into bundles containing a few isolated adipocytes [99] (Figure 4). Under correct regulation, claudins allow the permeation of huge cells, such as macrophages or lymphocytes, while blocking the passage of toxins produced by bacteria [100], thus supporting the role of claudins in tissue inflammation. In fact, it has been suggested that *CLDN5* is upregulated in extracellular vesicles isolated from the vitreous of individuals with T2D and diabetic retinopathy [74]. Adipocyte-induced fibrosis is also a process that occurs within AT in the obese condition [101]. In this context, using the liver as a model of chronic inflammation-associated fibrogenesis, CLDN1 was revealed to be a mediator and therapeutic target of tissue fibrosis, able to interact with ECM proteins and integrins [17]. In human organoid and mouse models, this claudin protein has been identified as a key player in the progression of liver fibrosis [17]. Moreover, monoclonal antibodies targeting non-junctional CLDN1 are currently being studied as anti-fibrotic treatments [17]. This evidence suggests the possibility of common mechanisms being present in other organs, such as AT, where CLDN1 could have a specific role in the development of fibrosis in obesity. Nonetheless, other studies have found that, while protein levels of CLDN1, CLDN3, and CLDN4 appeared to be elevated in patients with fibrotic lung disease, the mRNA levels of these claudins were not altered [102]. These contradictory results underscore the need for more scientific research in this area to be able to establish TJs and claudins as specific targets for the treatment of fibrosis. In a recent study published by our laboratory, we confirmed the high levels of CLDN1 in the VAT of patients living with obesity [103]. Moreover, the results of this study suggested that CLDN1 is specifically expressed in the visceral fatty depot, thus showing that its function in this tissue may be related to the location of the VAT in the omental cavity. According to this evidence, *CLDN1* was revealed to be a target gene induced in the omental AT of humans living with obesity and is associated with inflammation and fibrosis in this depot [103]. Moreover, *CLDN1* was found to be specifically upregulated in VAT-infiltrated T cells from patients living with obesity, suggesting a role for CLDN1 in pathophysiological AT alterations during obesity [103]. Taking all of this into consideration, the necessity to investigate the role of claudins in AT inflammation and fibrosis seems to be important to better understand AT physiology and function. This research could be key in supporting other studies that are currently trying to counteract the effects of obesity in the organism by immunotherapy [104]. In this context, this review aims to open the door to exploring the direct effect of anti-obesity treatments on claudin modulation within AT in the obesity context.

In response to whole-body energy imbalance, AT expands via the coordinated growth of the vasculature network to ensure the adequate perfusion of nutrients, hormones, and oxygen to the tissue [105]. Nevertheless, individuals living with obesity have been described to develop dysfunctional endothelial cells, contributing to inappropriate AT vascularization, resulting in suboptimal capillary density [106] (Figure 4). With this consideration in mind, TJs are crucial for the formation of the internal surface of blood and lymphatic vessels by endothelial cells, and TJ proteins are linked to angiogenesis. Indeed, TJs, including CLDN5, are critical determinants of the composition and integrity of endothelial barrier formation and function [107]. Nonetheless, the canonical and non-canonical functions of claudins could be orchestrating the inappropriate vascularization during AT angiogenesis in obesity; however, further studies in this field are necessary for a better understanding of this milestone.

Because of the adipocyte hypertrophy and inappropriate AT vascularization in obesity, adipocytes also generate a barrier limiting the amount of oxygen available to them [106]. This local hypoxia in obese AT could result in dead and necrotic adipocytes, leading to the recruitment of pro-inflammatory macrophages (able to engulf lipid droplets from dying adipocytes), which appear surrounding dead adipocytes in crown-like structures [108] (Figure 4). Generally, these macrophages are infiltrated as monocytes from the circulation, and they are located around the hypertrophic adipocytes which express monocyte chemoattractant protein-1 (MCP-1), among others [108]. Furthermore, pro-inflammatory macrophages accumulated in obese AT and local levels of cytokines, such as IL-6, TNF-α, and IL-1β, are upregulated by hypertrophic and/or necrotic adipocytes, further promoting macrophage infiltration into the fat depot [109] (Figure 4). Moreover, hypoxia-inducible microRNA-155 (post-transcriptional modulator) has been shown to suppress the expression of CLDN7 in eosinophilic esophagitis [110]. This evidence supports that obesity-induced hypoxia in AT may act through the dysregulation of several claudins. The role of claudin proteins in macrophages has not been extensively documented; nonetheless, a potential role has been described for CLDN1, CLDN2, and CLDN11 in anti-inflammatory macrophages from tumors [111]. This could suggest that these claudins exert opposing or overlapping effects, exhibiting differential or similar expression levels in pro-inflammatory macrophages. Moreover, hypoxia has been proven to affect CLDN expression. Specifically, CLDN1 contains hypoxia response elements (HREs) in its promoter region, and studies identified CLDN1 as a transcriptional target of hypoxia-inducible factor (HIF) in intestinal epithelia [112]. In this line, CLDN5 has also been found to be related to hypoxia by affecting autophagy in response to early hypoxia situations in endothelial cells [113]. Furthermore, *CLDN5* expression can be altered due to hypoxic conditions, thus resulting in disruption of the barrier function [114]. All these results support that the canonical and non-canonical functions of claudins could be orchestrating AT biology through key signaling pathways.

### 4.2. Claudins in Several Adipose Tissue-Related Signaling Pathways

PI3K/AKT is the main pathway by which claudins have been observed to participate in [115,116,117,118]. This pathway is necessary for energy metabolism and is a proven key regulated hub, which may be beneficial for the treatment of obesity [119]. When the PI3K/AKT pathway is dysregulated in obesity, it leads to abnormal AT expansion and chronic low-grade inflammation [119]. PDK1 is a mediator of the PI3K/AKT axis [120]. CLDN18 is reported to be capable of inhibiting PDK1 and AKT phosphorylation, thus halting cell proliferation in human lung adenocarcinoma cells [118]. Specifically, CLDN18 decreases PDK1 nuclear accumulation, leading to the disinhibition of the G1-S cell cycle stage provoked by nuclear PDK1 [118]. Moreover, CLDN1 and CLDN4 are essential players in the proliferation of esophageal squamous cells through the PI3K-AKT-mTOR pathway by feedback-regulating HIF-1α [116]. In this case, CLDN1 and CLDN4 are direct targets of HIF-1α under hypoxic conditions but can feed back regulate under conditions of normoxia [116].

Other claudins have been identified as participants in the adenylate cyclase (AC) pathway [19], which is part of PKA signaling and plays a predominant role in obesity and the maintenance of metabolic health [121]. The activation of AC by forskolin leads to the activation of specific protein phosphatases that dephosphorylate CLDN2, leading to its nuclear localization. This nuclear location allows CLDN2 to form a complex with other proteins, such as ZONAB—a Y-box transcription factor that is regulated by the ZO-1 protein [122]—and cyclin D1, enhancing cell proliferation [19]. Moreover, PKA phosphorylation also affects the subcellular location and function of CLDN1 in melanoma cells [123]. Furthermore, the PKA-STAT3 pathway has been found to regulate the expression of CLDN14 in rats through the calcium-sensing receptor (CaSR) [124]. CLDN9 and CLDN17 are also mediators of STAT3 regulation in hepatocytes [125,126]. Also, the JAK1/STAT1 axis is an example of a metabolic route controlled by claudin proteins; CLDN10 can enhance it in some cancerous cells [127]. The STAT proteins are a family of transcription factors that are directly related to the development of obesity as a result of the pathophysiological roles of some of these proteins in lipid and glucose metabolism [128]. More specifically, STAT3 has been widely studied due to its potential as a therapeutic target for the treatment of obesity, and its inactivation in AT leads to reduced adipose mass in mice [129].

Extracellular signal-regulated kinase (ERK) regulates fundamental cellular processes in response to extracellular stimuli [130]. Within AT, ERK is necessary to initiate preadipocyte differentiation and adipocyte maturation [131]. Moreover, this kinase is implicated in the development of obesity-related IR and T2D [132]. In this context, CLDN3 has been identified as a signaling mediator in the TNF-α pathway (via ERK1/2/slug), which modulates paracellular permeability in submandibular glands [133]. These data suggest that, in addition to canonical channel or barrier functions, claudins may have indirect effects on permeability via their interaction with different signaling pathways. What seems to be certain is that ERK signaling can be regulated in various ways: it can be repressed in some specific cases by CLDN6 in breast cancer cells or promoted by CLDN8 in colorectal cancer cells [134,135]. CLDN1 is also in the middle of a bidirectional signaling cascade with ERK, with several studies having indicated that CLDN1 affects ERK signaling and vice versa [136,137], thus, once again, showing the potential of claudins to influence key intracellular signaling pathways. In this manner, the role of CLDN1 is so essential in TNF-α/NFκB signaling that inhibition with a specific anti-CLDN1 antibody reverses the induction of this pathway in liver cells [17]. In this same study, CLDN1 was found to interact with EpCAM, the epidermal growth factor receptor (EGFR), and integrin subunit alpha 5 (ITGA5) [17]. ITGA5 is an important regulator of adipogenic differentiation in human adipose-derived stem cells, and this protein is a target of leptin in white AT in mice [138,139]. This, once again, emphasizes that the non-canonical role of claudins is of great importance for cell differentiation in the adipose depot. Moreover, CLDN1 has been recently targeted as a key player in the SRC/β-catenin pathway in some types of cancer. The possibility of claudin proteins as mediators of the SRC signaling hub is of great interest in the context of obesity, as SRC kinases have been reported to regulate fatty acid oxidation, promote human adipogenesis, and control energy balance between white and brown AT [140,141,142].

Claudins, as mentioned above, contain a PDZ-binding motif allowing them to interact with PDZ proteins, leading to the conformation of signaling complexes [31]. In this context, ZO-2 has been found to bind to the YV terminal sequence of claudins 1 to 8 through their PDZ domain [143]. In turn, ZO-2 interacts with the nuclear scaffolding factor SAF-B, a DNA-binding protein involved in RNA processing, thus directly relating claudins with gene regulation [31,144]. In fact, SAF-B1 is associated in vivo with PPARγ, and its expression changes during the early stages of adipogenesis [145]. ZO-3 also interacts with multiple signaling pathways, such as Akt in mouse models, where Akt phosphorylation can rescue ZO-3 expression [146].

All this scientific evidence highlights the importance of assessing the potential involvement of claudins in signaling pathways in other pathologies (e.g., metabolically related diseases) and even in healthy cell functioning, areas of study that have only recently been undertaken. The growing evidence on the non-canonical functions of claudins also strengthens the requirement for further studies on the involvement of claudins in cellular pathophysiology. In the context of obesity, one hypothesis may be that the up- or downregulation of claudins is related to inflammatory cell activation driven by non-epithelial cells. However, to confirm the role of these proteins in cellular processes, studies using knockout mice/cells and/or specific claudin up- and downregulation are needed. Taken as a whole, current data suggest that TJs—specifically the claudin family—may be novel molecular actors with an important role in AT physiology, disease, and remodeling during obesity, with a distinctive role in several signaling pathways.

## 5. Conclusions and Perspective

Defining the role of claudins in complex intracellular signaling pathways is increasingly catching the scientific eye. While the involvement of these proteins in pathologies such as cancer has been extensively studied, the consequences of the differential regulation of claudins in complex metabolic processes have been less studied, if at all. However, recent data have strongly indicated their potential role in AT pathophysiology in obesity and perhaps in associated metabolic disorders. Furthermore, due to the presence of claudins in cell membranes, this field of study could answer some of the most critical processes that accompany the development of obesity, such as AT permeability and immune cell infiltration. Moreover, the new perspective of considering claudins not only as TJ proteins but also as cytoplasmic proteins and, therefore, actors in intracellular signaling, or even in the regulation of gene expression, opens a broad, new, interesting perspective on metabolically related disorders.

Additionally, the fact that there are few studies specifically examining the role of claudins in AT, despite evidence of their important role in obesity and associated pathologies, makes research aimed at understanding the mechanisms of action of claudins in the adipose depot especially necessary.

## Figures and Tables

**Table 1:** Canonical function of claudins. The different canonical functions of claudins in tissue permeability. Adapted from Krug et al. (2012) [32] and Tsukita et al. (2019) [14], and Meoli & Günzel (2023) [33]. The (?) indicates that the exact role in ion permeability is still unclear.

Claudin	Ion Permeability	Function
CLDN1	Ions/Water	Barrier-forming
CLDN2	Cations/Water	Channel-forming
CLDN3	Ions/Water	Barrier-forming
CLDN4	Cations (?)	Barrier/channel-forming
CLDN5	Ions/Water	Barrier-forming
CLDN6	Ions/Water	Barrier-forming
CLDN7	Anions (?)	Barrier/channel forming
CLDN8	Anions/Cations	Barrier/channel forming
CLDN9	Ions/Water	Barrier-forming
CLDN10a	Anions	Channel-forming
CLDN10b	Cations	Channel-forming
CLDN11	Ions/Water	Barrier-forming
CLDN12	Cations	Barrier/channel forming
CLDN14	Ions/Water	Barrier-forming
CLDN15	Cations/water	Channel-forming
CLDN16	Anions/Cations	Channel-forming
CLDN17	Anions	Channel-forming
CLDN18	Ions/Water	Barrier-forming
CLDN19	Anions/Cations	Barrier-forming

**Table 2:** Effect of claudin proteins on signaling pathways. The different claudin proteins and the effect they have on the pathway and/or the transcription factor they regulate (upregulation or downregulation), and the most common cell type where this effect has been studied.

Claudin	Cellular Pathways and/or Transcription Factors	Effect	Cell Type
CLDN1	Ras/Erk; Src/AKT; NF-κβ; PI3K/AKT; Src	**↑**	Intestine, epithelial, colon, liver, esophageal squamous cells
CLDN2	AC; FOXO	**↑**	Epithelial cells, lung adenocarcinoma
CLDN3	ERK	**↑**	Submandibular glands
CLDN4	TGFβ/Smad; PI3K/AKT	**↑**	Lung, esophageal squamous cells
CLDN5	Wnt	**↑**	Podocytes, endothelial cells
CLDN6	ERK	**↓**	Breast cancer
CLDN7	Wnt; Src/AKT	**↑**	Epithelial, intestine
CLDN8	ERK	**↑**	Colorectal cancer
CLDN9	STAT3	**↑**	Hepatocytes
CLDN10	JAK1/STAT1	**↑**	Osteosarcoma
CLDN11	Notch	**↑**	Osteoblasts
CLDN17	STAT3	**↑**	Hepatocytes
CLDN18	PDK1/AKT	**↑**	Lung adenocarcinoma

## Abbreviations

Adenylate cyclase (AC); adherens junction (AJ); adipose tissue (AT); Ak strain transforming (AKT); blood–brain barrier (BBB); claudin protein (CLDN); claudin gene (*CLDN*); extracellular matrix (ECM); epidermal growth factor receptor (EGFR); extracellular signal-regulated kinases (ERKs); epithelial cell adhesion Molecule (EpCAM); Forkhead box O (FOXO); insulin resistance (IR); integrin subunit alpha (ITGA); Janus kinase (JAK); junctional adhesion molecule (JAM); MAL and related proteins for vesicle trafficking and membrane link (MARVEL); lipopolysaccharides (LPSs); nuclear factor kappa B (NFκB); Pyruvate Dehydrogenase Kinase 1 (PDK1); Phosphatidylinositol-4,5-Bisphosphate 3-Kinase (PI3K); protein kinase A (PKA); protein kinase C (PKC); peroxisome proliferator-activated receptor gamma (PPARγ); subcutaneous adipose tissue (SAT); proto-oncogene c-Src (SRC); Signal Transducer and Activator of Transcription (STAT); triglycerides (TG); Transforming Growth Factor beta (TGFβ); tight junction (TJ); tight junction protein gene (*TJP*); Tumor Necrosis Factor alpha (TNF-α); visceral adipose tissue (VAT); World Health Organization (WHO); Zonula Occludens (ZO).
